# Paraneoplastic ocular proptosis secondary to a pulmonary benign metastasizing leiomyoma: a case report and brief literature review

**DOI:** 10.1093/jscr/rjag081

**Published:** 2026-04-30

**Authors:** María Alejandra Amaya, Julio César Granada Camacho, Luis Gerardo García-Herreros Hellal, Juliana Pardo

**Affiliations:** Faculty of Medicine, Universidad del Bosque, Av. Cra. 9 No. 131 A - 02, Bogotá 111321, Colombia; Thoracic Surgery Department, Fundación Santa Fe de Bogotá, Calle 119 # 7-75, Bogotá 110111, Colombia; Thoracic Surgery Department, Fundación Santa Fe de Bogotá, Calle 119 # 7-75, Bogotá 110111, Colombia; Faculty of Medicine, Universidad de los Andes, Cra 1 Nº 18A- 12, Bogotá 111711, Colombia

**Keywords:** paraneoplastic syndrome, proptosis, lung neoplasm, leiomyoma

## Abstract

Paraneoplastic syndromes are indirect clinical manifestations of cancer, arising from immunological mechanisms or tumor-derived substances. The most common paraneoplastic syndromes associated with lung cancer are endocrine syndromes. We report the case of a 50- year-old woman with progressive ocular proptosis and orbital pain who was evaluated by multiple specialties with various diagnostic tests that did not explain her symptoms. She received immunosuppressive therapy without clinical improvement. A right paracardiac mass was subsequently identified on imaging, initially suspected to be a pericardial cyst, but prior studies over 3 years showed significant interval growth. The lesion was resected via thoracoscopic surgery, and the patient showed symptomatic improvement within a few days. Pathological results revealed a benign metastasizing leiomyoma. Given clinical presentation and postoperative evolution, a paraneoplastic mechanism is considered the most likely cause of the patient’s ocular proptosis.

## Introduction

Paraneoplastic syndromes are a set of clinical manifestations caused by cancer, which are not explained by direct tumor invasion, mass effect, or metastasis, but result instead from immunological mechanisms or tumor-derived biochemical substances. They affect up to 8% of people with cancer [1]. The main types of cancer most frequently associated with paraneoplastic syndromes are lung tumors, breast cancer, gynecological, and hematological neoplasms [2]. When referring to lung tumors, endocrine syndromes are the most common ones, although patients may also present neurological, musculoskeletal, or cardiovascular syndromes, among others [3].

We report the case of a 50-year-old woman with paraneoplastic ocular proptosis secondary to a benign metastasizing leiomyoma involving the lung.

## Case report

A 50-year-old woman with a medical history including hysterectomy for abnormal uterine bleeding, presented with progressive ocular proptosis for 20 months, associated with orbital pain, without any other symptoms. She was evaluated by endocrinology, orbital specialist, neurology, rheumatology, and infectious, autoimmune, and thyroid causes were excluded through appropriate testing without abnormal findings. Additionally, brain magnetic resonance imaging (MRI) was normal, and orbital MRI showed only increased retro-orbital fat. Due to persistent symptoms, treatment with betamethasone was started, without any clinical improvement.

**Figure 1:**
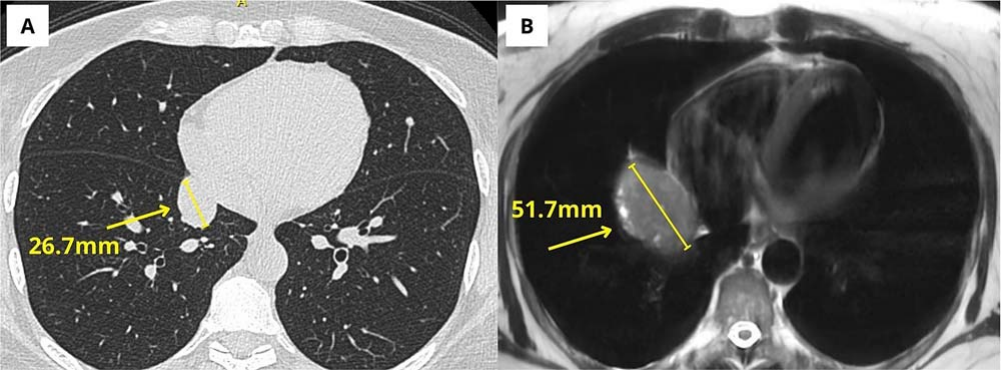
(A) Chest computed tomography from February 2022, showing a right paracardiac lesion reported as a pericardial cyst. (B) Chest MRI from July 2025 showing the same lesion as a well-defined oval mass with clear interval growth.

During follow-up, an abdominal MRI was performed to evaluate a hepatic cyst and revealed a right paracardiac mass, prompting referral to the thoracic surgery department. Review of imaging over the prior 3 years showed that the lesion had initially been identified as a pericardial cyst, but it demonstrated significant interval growth and developed radiologic features consistent with a carcinoid tumor (Fig. 1).

**Figure 2:**
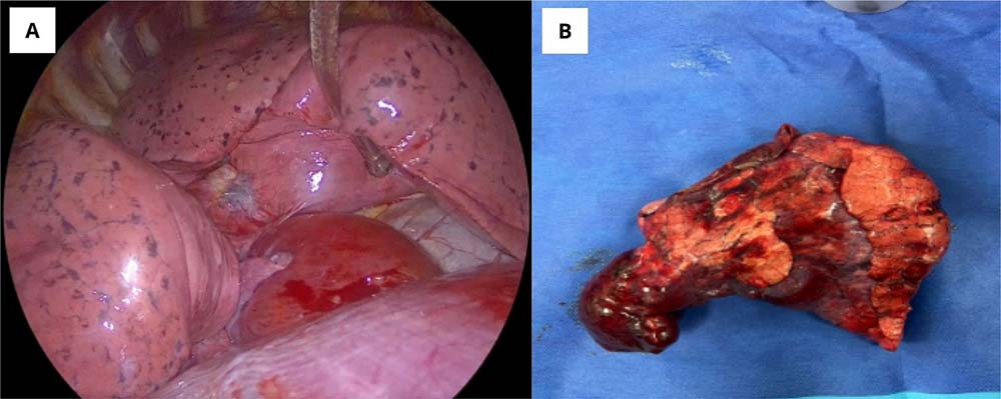
(A) Intraoperative image of the tumor *in situ*. (B) Resected tumor specimen.

**Figure 3:**
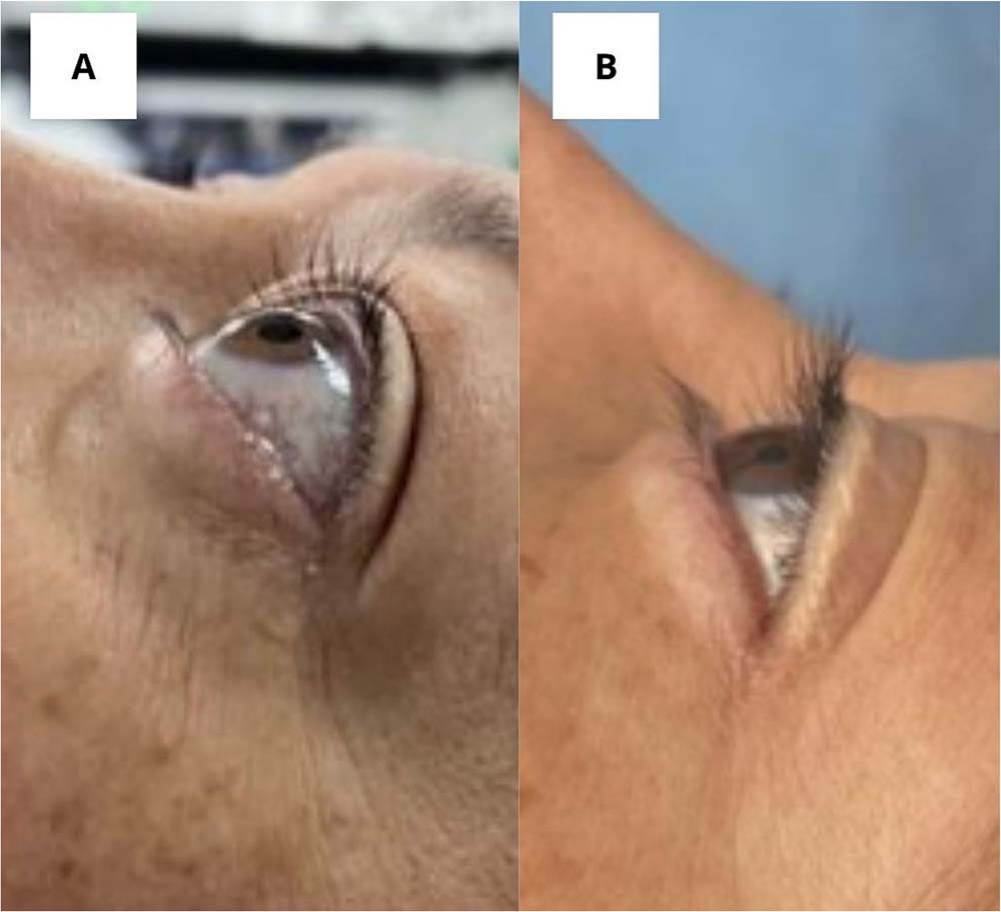
(A) Photograph taken before surgery. (B) Photograph taken 2 weeks after surgery.

A right lower lobectomy with mediastinal lymph node dissection was performed via video- assisted thoracoscopic surgery without complications. Fig. 2A shows the tumor *in situ* and Fig. 2B shows the resected specimen. After the procedure, the patient had a favorable postoperative evolution, and the ocular proptosis began to diminish within a few days. Fig. 3A depicts an image before the surgery, and Fig. 3B demonstrates the patient’s progress 2 weeks later, with evidence of a significant decrease in ocular proptosis and improvement in the previously reported orbital pain.

Despite the initial hypothesis, histopathological analysis reported intrapulmonary involvement by a benign smooth-muscle mesenchymal neoplasm consistent with benign metastasizing leiomyoma.

## Discussion

Paraneoplastic syndromes represent up to 10% of the clinical manifestations associated with lung tumors, with small-cell carcinoma being the most prevalent subtype [3, 4]. Endocrine paraneoplastic syndromes are the most frequently encountered, with humoral hypercalcemia of malignancy and the syndrome of inappropriate antidiuretic hormone secretion being the most common [4]. Nevertheless, ocular proptosis as a paraneoplastic syndrome is rare, and only six cases have been reported in the literature, six of them associated with lung tumors [5–10]. Reported lung tumors causing proptosis include pulmonary adenocarcinoma, pleomorphic carcinoma, small-cell lung cancer, and squamous cell carcinoma [5–10]. In our case, the tumor identified was a benign metastasizing leiomyoma.

Benign metastasizing leiomyoma is a rare disease characterized by the proliferation and metastasis of smooth muscle cells, usually originating from a uterine leiomyoma with metastatic growth at extra-uterine sites. It typically occurs in women with a history of uterine leiomyomas or prior hysterectomy [11]. To date, no cases have been reported in the literature describing paraneoplastic syndromes secondary to this type of tumor. The pathophysiology underlying the proptosis remains unclear, but it is believed that these cases may be caused by multiple mechanisms, such as immune cell infiltration, fibroblast proliferation, and abnormal immune activation triggered by inflammatory cytokines [9].

In all reviewed cases, ocular proptosis improved after treatment of the primary tumor. In our case, the patient had undergone an extensive diagnostic workup in an attempt to explain the ocular proptosis, as well as immunosuppressive treatment without clinical improvement. After surgical resection, the patient began to exhibit diminished ocular proptosis and orbital pain, supporting the hypothesis of a paraneoplastic syndrome as the cause of her symptoms.

## References

[1] Badawy M, Revzin MV, Consul N et al. Paraneoplastic syndromes from head to toe: pathophysiology, imaging features, and workup. Radiographics 2023;43:1–2. 10.1148/rg.220085 36795597

[2] Pelosof LC, Gerber DE. Paraneoplastic syndromes: an approach to diagnosis and treatment. Mayo Clin Proc 2010;85:838. 10.4065/mcp.2010.0099 20810794 PMC2931619

[3] Kanaji N, Watanabe N, Kita N et al. Paraneoplastic syndromes associated with lung cancer. World. J Clin Oncol 2014;5:197. 10.5306/wjco.v5.i3.197 PMC4127595 25114839

[4] Anwar A, Jafri F, Ashraf S et al. Paraneoplastic syndromes in lung cancer and their management. Ann Transl Med 2019;7:359–9. 10.21037/atm.2019.04.86 31516905 PMC6712246

[5] Kim DI, Lock G. Bilateral extraocular muscle enlargement and proptosis associated with squamous cell carcinoma of the lung. BJR Case Rep 2018;5:20180049. 10.1259/bjrcr.20180049 31131124 PMC6519496

[6] Romano LM, Besocke AG. Proptosis bilateral paraneoplásica asociada a carcinoma de pulmón. Rev Neurol 2009;49:389–90. 10.33588/rn.4907.2009147 19774537

[7] Diacon AH, Schuurmans MM, Colesky FJ et al. Paraneoplastic bilateral proptosis in a case of non-small cell lung cancer. Chest 2003;123:627–9. 12576392 10.1378/chest.123.2.627

[8] Mehta P, Chickadasarahally S, Hedley N et al. Extraocular muscle enlargement as a paraneoplastic effect of breast carcinoma in a male patient. Ophthalmic Plast Reconstr Surg 2011;27:e146–7. 10.1097/IOP.0b013e3182078e31 21283026

[9] Kuzunishi Y, Tsuzuku A, Asano F et al. Pleomorphic carcinoma with exophthalmos and a subsequent diagnosis of paraneoplastic syndrome. Intern Med 2021;60:605–9. 10.2169/internalmedicine.5286-20 32999228 PMC7946491

[10] Fukushima M, Hatanaka R, Tsushima T et al. A case of squamous cell carcinoma of the lung associated with exophthalmos and hypercalcemia. Kyobu Geka 1998;51:168–73. 9492473

[11] Reyes-Esparza A, Miranda-Castañón F, Amaya-Téllez M et al. Leiomioma metastatizante benigno: reporte de Caso. Gaceta mexicana de oncología 2022;21:123–7. 10.24875/j.gamo.22000023

